# Spotlight for healthy adolescents and adolescents with preexisting chronic diseases during the COVID-19 pandemic

**DOI:** 10.6061/clinics/2020/e1931

**Published:** 2020-04-30

**Authors:** Clovis Artur Silva, Lígia Bruni Queiroz, Claudia de Brito Fonseca, Luís Eduardo Vargas da Silva, Benito Lourenço, Heloisa Helena Sousa Marques

**Affiliations:** I Instituto da Crianca e do Adolescente (ICr), Hospital das Clinicas HCFMUSP, Faculdade Medicina, Universidade de Sao Paulo, Sao Paulo, SP, BR; II Unidade Pediatrica de Doencas Infecciosas, Instituto da Crianca e do Adolescente (ICr), Hospital das Clinicas HCFMUSP, Faculdade Medicina, Universidade de Sao Paulo, Sao Paulo, SP, BR

Recently, coronavirus disease (COVID-19) was declared a pandemic by the World Health Organization (WHO). The virus that causes the emerging infectious disease was designated by the International Committee on Taxonomy of Viruses as severe acute respiratory syndrome coronavirus 2 (SARS-CoV-2) 1,2.

COVID-19 has been reported predominantly in adult patients. The clinical, laboratorial, and radiographic abnormalities for adults with COVID-19 include fever, dry cough, myalgia, fatigue, chest discomfort, respiratory frequency >30/min, blood oxygen saturation <93%, leukopenia, lymphopenia, and chest radiographic or computed tomographic scan with mild to severe pneumonia 1-3. The most important risk factors or comorbidities in adult COVID-19 patients include: age more than 60 years, cardiovascular disease, diabetes mellitus, arterial hypertension, chronic respiratory disease, and cancer 2-4.

In contrast to adults with COVID-19, the majority of children and adolescents infected with SARS-CoV-2 exhibited milder disease and also presented with nasal congestion, rhinorrhea, pharyngeal erythema, diarrhea, and vomiting. These young patients showed an adequate treatment response and a short duration to COVID-19 resolution 5,6.

Adolescent populations have rarely been evaluated in the context of this emerging infectious disease. A recent study reported on 72,314 patients with COVID-19 from the Chinese Center for Disease Control and Prevention, 1% of whom were adolescents (10-19 years) 3. Notably, this infectious disease occurred in 169/4,212 (4%) adolescents in the Republic of Korea study 7.

Another Chinese study, including 2,143 pediatric patients, showed that 35% of the patients were adolescents. Disease severity was higher in infants than in other pediatric ages. Severe and critical cases were uncommon in adolescents, with an occurrence rate of 4% and 3% in the age groups 11-15 and ≥16 years, respectively 8.

Moreover, silent infection is defined as positivity for SARS-CoV-2, without any clinical manifestations or chest imaging abnormalities 9. Adolescent populations frequently present with asymptomatic SARS-CoV-2 infection, and these infected populations are the main source of SARS-CoV-2 infections in the pediatric age group 9,10.

Regarding the pathogenesis of COVID-19, one of the possible reasons for the lower prevalence of COVID-19 in children and adolescents than in adults is the lower expression of the angiotensin-converting enzyme 2 receptor (ACE-2) in the pediatric age group. This receptor is necessary for the entry of SARS-CoV-2 into the cells, particularly in the lung, heart, and intestine 11. ACE-2 expression may increase angiotensin II production, resulting in an increase in vasoconstriction and pulmonary vascular permeability 12. A recent meta-analysis showed that comorbidities may also be changed by epigenetic regulation, with increased expression of ACE-2 in human lung cells associated with severe COVID-19 13.

As previously mentioned, critically ill cases have been infrequently reported in adolescents. Adolescents with severe acute respiratory failure and multi-organ dysfunction syndrome may present signs and symptoms of cytokine storm syndrome, triggered by SARS-CoV-2. Indeed, this subgroup of adolescents with COVID-19 may present with high fever; confusion; anemia; thrombocytopenia; neutropenia; coagulopathy; hyperferritinemia; and elevated liver enzyme, D-dimer, lactate dehydrogenase, and soluble CD25 levels 14. An elevated cytokine profile associated with this complication mainly includes increases in interleukin (IL)-2, IL-7, granulocyte-colony stimulating factor, and tumor necrosis factor-alfa levels 15. Notably, these findings were similar to the findings in adolescents with familial hemophagocytic lymphohistiocytosis or macrophage activation syndrome due to systemic juvenile idiopathic arthritis and in systemic lupus erythematosus patients 16-18.

Furthermore, the prevalence of adolescents with preexisting chronic diseases has been rising in the last years, resulting in considerable morbidity and mortality. These patients may present high disease complexity and severity with an unpredictable course, requiring multiple medications, several physician appointments in outpatient clinics and emergency departments, several hospital admissions, and an undoubtedly high risk of infectious diseases. The most prevalent diagnoses of these chronic conditions in adolescents are asthma, obesity, cancer, diabetes mellitus, transplantation, and autoimmune diseases 19-21.

Thus, preexisting chronic conditions in adolescents with confirmed COVID-19 may be associated with the risk of progression to severe disease (acute respiratory failure and/or multi-organ dysfunction syndrome) and death 22. Despite recent publications, the real predisposition and risk of COVID-19 in adolescent populations with preexisting chronic conditions are still unknown.

In this regard, this emerging infectious disease appears to have a milder course and a less aggressive attack rate in the pediatric population with asthma 23. In contrast, obese patients may be at a higher risk of impaired outcomes associated with COVID-19 24. Chronic hyperglycemia may impact the immune function and also increases the morbimortality risk due to this acute infectious disease 24. The long-term effects of this global health emergency, with school closure, physical inactivity, sedentary behavior, and consumption of calories-dense comfort foods, may increase the risk of weight gain in teenagers, particularly those living in urban districts 25-27.

The data of immunocompromised children and adolescents with cancer and COVID-19 are scarce 28-29, as well as in adult cancer patients 4. The majority of childhood cancers are aggressive, requiring prompt and intensive multiagent chemotherapy or stem cell transplantation, and postponing these treatments are not indicated during this pandemic 28-29.

In addition, there are limited data regarding COVID-19 in adolescents and adults with preexisting chronic conditions, particularly those with autoimmune rheumatic diseases 2,12,, inflammatory bowel disease 33, autoimmune hepatitis, and those undergoing liver transplantation 34. Patients with these chronic diseases have particular concerns with regard to their risk of severe infection and to the management of their immunosuppressive or immune-modifying drug treatments during the pandemic 35. However, recent data do not indicate an increased risk of SARS-CoV-2 infection in patients with these chronic conditions 30-35.

It is notable that the recent Bergamo report showed that none of the patients who underwent liver transplantation had severe COVID-19, despite 3/700 (0.4%) having tested positive for SARS-CoV-2 34. Another Italian case series showed that 13/320 (4%) adult patients with chronic arthritis under immunosuppressant agents had suspected or confirmed COVID-19. Only one (0.3%) patient required hospitalization for oxygen supplementation, and none of them died 36.

Since the pandemic has rapidly deteriorated into a global crisis, several national and local public health authorities and international agencies (particularly the WHO) 37, have regularly provided updated COVID-19 recommendations. Some additional recommendations are suggested for healthy adolescents and adolescents with chronic illnesses during COVID-19:

1. There are six WHO advocacies for parents and adolescents during confinement: 1) one-on-one time (including talking about sports, cooking, and physical activities), 2) staying positive in difficult times, 3) creating a daily routine, 4) avoiding bad behavior and habits, 5) keeping calm and managing stress, and 6) being honest and supportive when talking about COVID-19 37.
2. Adolescents should be encouraged to practice social distancing to limit potential exposure and to wear masks to protect against SARS-CoV-2, particularly outside of the home and during outpatient visits.
3. Telemedicine seems to be an effective and sustainable technology for adolescents, parents, and multidisciplinary teams. This technology may help conduct online appointments with respect to adolescent physicians and pediatric subspecialties during the COVID-19 pandemic 38-40.
4. Adolescence is a transitional stage involving biological, psychological, and cultural expressions and cognitive development. During this period, teenagers gradually become more independent, gaining autonomy from parents, bonding with their peers, and beginning romantic interests 41,42. Feelings, fear of developing the coronavirus infection and dying, peer relations, closing of schools and universities, suspending sporting events, familial financial loss, domestic conflict, parents' tension, and novel daily hygiene habits should be discussed in the adolescent appointments. These appointments may be focused on only teenagers or may include adolescents and their parents 43,44.
5. A home schooling plan for adolescents should be applied by schools/universities and teachers using online tools 44.
6. Healthy foods should be reinforced during quarantine, avoiding ultra-processed foods, reinforcing cooking, and stocking homemade foods.
7. Physical exercise is essential during the lockdown. There are several specialized online channels with recreational activities and easy access via the internet for adolescents who stay at home. Outdoor activities should be avoided.
8. Quarantine has been a necessary preventive measure during this pandemic around the world and may result in negative psychological outcomes in adolescents and their families. Therefore, information and communication for adolescents (using telemedicine or telephone support lines) and motivation through social networking by mobile phone should be priorities 45. Psychological and psychiatric issues (such as acute stress, anxiety, depression, post-traumatic stress disorder, and emotional exhaustion) may occur or worsen in healthy adolescents and adolescents with chronic illnesses during the COVID-19 quarantine/lockdown and social isolation, requiring online mental health services (as cognitive behavioral therapy) 39,40,45.
9. Home confinements may induce longer screen time and sleep issues. Regular sleep patterns and parental control of screen time should be reinforced during the lockdown.
10. Drinking alcohol does not protect against COVID-19 and alcohol intake can increase the risk of health issues in adolescents 37.
11. Since prisons are the epicenters for infectious diseases, the prevention and control of COVID-19 are required for specific adolescents and youth populations in jail, including individual protection measures, social distancing, regular environmental cleaning, and limiting prison visits 46,47.
12. During the pandemic, routine appointments and elective surgeries and procedures should be postponed for adolescents.
13. The number of subjects visiting adolescents with chronic diseases must be reduced in outpatient clinics, day-clinics, and hospitals during the pandemic, especially for those taking immunosuppressive agents or for pediatric transplantation patients 29.
14. Adherence to medications should be reinforced for all adolescents with preexisting chronic disease 42.
15. Immunosuppressive and immune-modifying drug treatments, multiagent chemotherapy, and stem cell transplantation should be evaluated for each adolescent without COVID-19, according to their specific preexisting chronic disease.
16. Medications should be adjusted or stopped for each adolescent who has been infected with for SARS-CoV-2 or has been confirmed as having COVID-19 for each specific preexisting chronic condition. The immunosuppressive agents may be restarted after two weeks if the adolescent has not developed COVID-19 manifestations or after complete sign and symptom resolution, as suggested for inflammatory bowel disease patients 35.
17. The comorbidities in adolescents that are associated with a high risk of severe COVID-19 in adult populations should be identified, stopped, and/or treated: smoking, arterial hypertension, diabetes mellitus, asthma, and chronic kidney disease 2-4.
18. The vaccination card must be updated. During the COVID-19 pandemic, adolescents with preexisting chronic diseases should receive the annual influenza vaccine, regardless of current disease activity and the use of any immunosuppressive drug, as suggested for adolescents with autoimmune rheumatic diseases 48. The adolescents in prison should also receive this annual immunization.
19. Adolescents with autoimmune diseases, who have confirmed COVID-19 must be strictly monitored for the possible risk of reactivation of the disease after the resolution of this viral infection 32.
20. Owing to the negative economic impact of this pandemic, vulnerable adolescents and their families should receive financial support.

In conclusion, spotlight and recommendations for healthy adolescents and adolescents with preexisting chronic diseases during the COVID-19 pandemic should be reinforced for all subjects, particularly for those under quarantine/lockdown. Further studies will be necessary to clarify and to assess specific adolescent populations during this emerging infectious disease pandemic.

## AUTHOR CONTRIBUTIONS

Silva CA conceived the study, was responsible for the data curation, funding acquisition, investigation, methodology and project administration. Queiroz LB, Fonseca CB, Silva LEV, Lourenço B and Marques HHS conceived the study, were responsible for the data curation, formal analysis, investigation, methodology and project administration.
